# Sonographic findings in scabies

**DOI:** 10.1007/s40477-022-00700-4

**Published:** 2022-08-26

**Authors:** M. Dominguez-Santas, G. Roustan-Gullon, F. Alfageme-Roldan

**Affiliations:** 1 Dermatology Department, Ramon y Cajal University Hospital, Carretera Colmenar Viejo km 9.100, 28034 Madrid, Spain; 2 Dermatology Department, Puerta de Hierro University Hospital, Madrid, Spain

**Keywords:** Scabies, Ultrasound, Dermatology

## Abstract

Scabies is a cutaneous infestation caused by *Sarcoptes scabiei var. hominis*, a small mite that performs its whole life cycle within the epidermis. In this case report, we provide images of the sonographic signs of scabies. We found that the adult mite can be seen as a hyperechoic well-defined ovoid area within the epidermal layer at the end of the hypoechoic burrow, while the eggs correspond to tiny heteroechoic dots along the burrow. In conclusion, ultrasound may prove useful to differentiate between inhabited vs non-inhabited scabiotic burrows.

## Introduction

Scabies is a skin condition caused by the infestation with *Sarcoptes scabiei var. hominis*. It presents as a very pruritic exanthem, with erythematous papules and excoriations. Since many other skin conditions can be pruritic, the diagnosis is often made by observing the burrow made by the female mite. This burrow though, is not always visible in all patients with scabies. Burrows can be easily confused with the scratches caused by pruritus, even with the aid of dermoscopy. Thus, ultrasound could prove a useful tool in its diagnosis by helping to recognize mites in the epidermal layer [1, 2].

## Case report

A 51-year-old man presented to our Dermatology department with a history of very pruritic skin lesions that had appeared 1 month prior to consultation. He lived alone. No new drugs were initiated the months prior to this episode, and the only drug he episodically took was acetaminophen for headaches. He reported that pruritus worsened during night and affected the whole body, sparing the head.

**Fig. 1:**
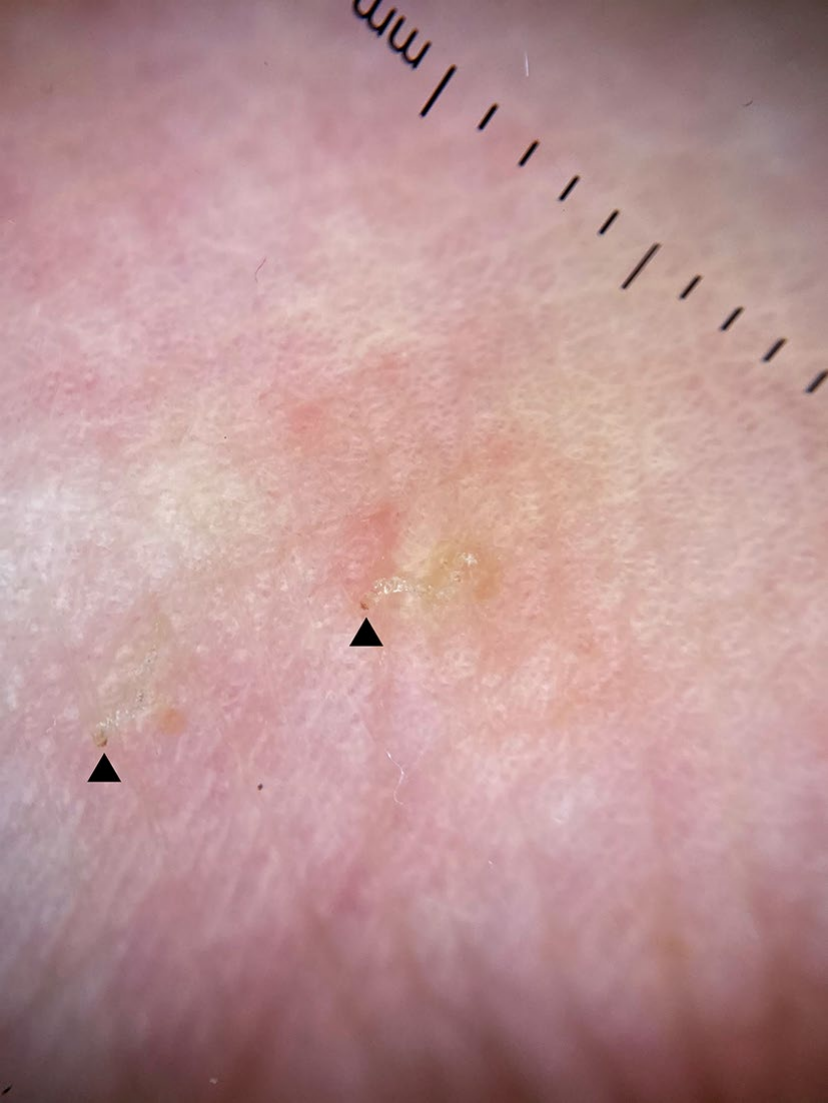
Dermoscopy of scabiotic burrow with the “jet with a contrail” sign and the “triangle” sign. Arrows mark the “triangle” sign, pointing out the exact location of the mite

Dermatological examination showed erythematous papules in trunk, legs and arms. Erythematous nodules were present in the scrotum. Mucosae were not affected. With dermoscopy, a scabiotic burrow with the “jet with a contrail” sign and the “triangle” sign was seen in the volar aspect of the left wrist (Fig. 1). We then performed an ultrasound exam with a variable frequency 10–22 MHz lineal probe attached to a MyLab^TM^9 platform (Esaote). A copious amount of gel was used, and the probe was placed longitudinally along the burrow that we had previously identified with dermoscopy.

**Fig. 2:**
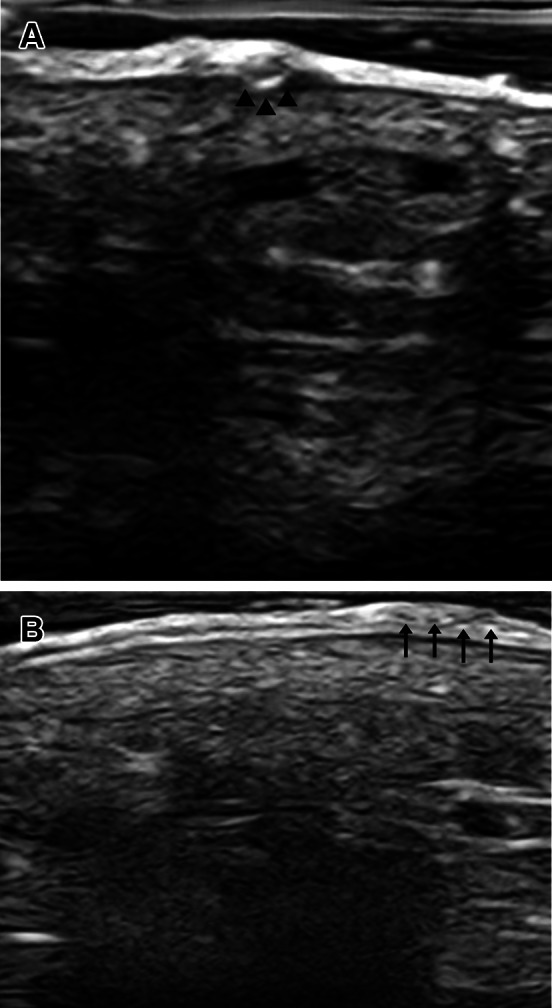
Greyscale US exam. **A** Hyperechoic well-defined ovoid area (marked with arrows) within the epidermal layer at the site where the “triangle” sign is present. **B** Tiny heteroechoic dots within epidermis

We could observe in the site with the “triangle” sign a hyperechoic well-defined ovoid area within the epidermal layer (Fig. 2A). The burrow showed an image of tiny heteroechoic dots within the epidermis that, we could hypothesize, were the eggs laid by the mite (Fig. 2B).

## Discussion

Scabies is a skin disorder that severely affects patients due to intense pruritus. It is sometimes misdiagnosed due to the inability of correctly recognizing scabiotic burrows, as they are easily mistaken with scratches caused by other pruritic conditions [1, 2].

Dermoscopy helps us differentiate some of these scratches from actual burrows, as a burrow can be seen as a wavy, hyperkeratotic, greyish-white tunnel-like structure [3–5].

The term “jet with a contrail” sign refers to a scabiotic burrow seen in dermoscopy with a black triangle at one end. This black triangle is also called the “triangle” sign, it is a pathognomonic sign of scabies and allows us to make a diagnosis without the need for a biopsy or a skin scraping. It also points out the exact location of the female adult mite. But not every burrow can be correctly recognized with dermoscopy alone, as many of them may lack this sign or other dermoscopic criteria [3–5].

With the aid of sonography, we could correctly assess those dermoscopically-unspecific burrow-like structures, to verify the presence or absence of adult mites.

This is not only useful as a means to perform a correct diagnosis, but also for assessing that treatment was performed correctly. After a correct treatment and resolution of the disease, pruritus can persist for a month [6], making the patient believe that the mite was resistant to the therapy. With ultrasound exam, we could demonstrate the absence or presence of real mites within the epidermis of non-specific burrow-like scratches.

There are no previous reports on high frequency ultrasound of scabies as far as we know. We propose high frequency ultrasound, as a non-invasive, new, complementary diagnostic method in scabies in cases in which clinical examination or dermoscopy are non-conclusive.

## Declarations

### Human and animal studies

All human and animal studies are approved by an Institutional Review Board.
